# An infrared night vision image enhancement algorithm based on cross-level feature fusion

**DOI:** 10.1371/journal.pone.0330349

**Published:** 2025-09-04

**Authors:** Xuanming Wang

**Affiliations:** School of Information and Communication Engineering, Beijing University of Posts and Telecommunications, Beijing, China; University of Baghdad, IRAQ

## Abstract

Infrared night vision images are caused by color overflow and coloring discontinuity due to insufficient light at night, resulting in larger halo area and lower PSNR value after enhancement by single feature fusion method. For this reason, an infrared night vision image enhancement algorithm based on cross-level feature fusion is proposed. This method is used to denoise infrared night vision images, based on smooth wavelet decomposition. By labeling image edges and noise, and utilizing neighborhood based wavelet coefficient shrinkage algorithm, the noise interference in the image is effectively reduced; preliminary enhancement was performed on the denoised image, using Retinex algorithm combined with bilateral filtering method to estimate illuminance, and Sigmoid function was used to enhance the reflection area, improving the overall visual effect of the image. Based on the principle of cross-level feature adaptive fusion, a cross-level feature fusion network is constructed to further enhance the feature information of the infrared night vision image through the steps of multi-level feature extraction, feature reconstruction and adaptive cross-level feature fusion, and the output of the model is optimized by using the joint loss function, which realizes the high-quality enhancement of the infrared night vision image. The experimental results show that when the method is utilized for infrared night vision image enhancement, the PSNR is higher than 30dB, the SSIM is higher than 0.73, and the enhancement effect is good and the performance is high.

## 1 Introduction

Infrared imaging technology, as an important component of modern optoelectronic technology, is widely used in various fields such as night monitoring, medical diagnosis [1], building inspection, and environmental monitoring. Infrared image is formed by capturing the infrared radiation emitted by the target object itself [2], which can reflect the temperature distribution of the object, thus realizing the detection and identification of the target. However, due to the characteristics of the infrared imaging system and the influence of the external environment, infrared images often have low contrast, low signal-to-noise ratio, fuzzy edges and other problems, which seriously affect the subsequent processing of infrared images and the application of the effect [3–4].

In order to optimize the effect of infrared images, the reference [5] method firstly decomposes the infrared images into low-frequency and high-frequency images based on the Retinex with improved bootstrap filtering, in order to make full use of the dynamic space at the pixel level, the low-frequency images are uniformly redistributed to improve the brightness and clarity of the images; and then edge extraction is performed on high-frequency images using directional gradient operators, followed by edge enhancement to further improve the contrast of the images. The enhanced low-frequency and high-frequency images are retinex inverse transformed to obtain the enhanced infrared images. In the reference [6], a 16-bit image is obtained by effective feature extraction of 14-bit infrared image with automatic linear mapping, which improves the image visualization effect; then the Generalized Unsharp Masking (GUM) algorithm is introduced with Multi-Scale Retinex with Color Restoration (MSR) to enhance the contrast of the image. Retinex with Color Restoration (MSRCR) enhancement algorithm, the effective information of different scales of the image is obtained, and the contrast of the image is improved; finally, the adaptive weight map is designed, and combined with the characteristics of the image pyramid structure, the effective information of the different feature layers is fused to enhance the brightness of the image, and the texture of the image is enriched. The image brightness is enhanced and the texture information is enriched. Reference [7] method firstly, in order to overcome the problem of infrared image degradation caused by the fixed scale parameter and light scattering, the atmospheric transmittance is utilized to obtain the full-scale mapping map of the Retinex scale parameter, so as to effectively improve the clarity of the image, and the input image and the input image processed by using the full-scale Retinex are taken as the first and second inputs of the algorithm; secondly, to solve the problems of artifacts and detail loss in traditional wavelet threshold functions during image denoising, an improved wavelet threshold function is designed. By introducing a scale factor, after calculating the wavelet coefficients of each high-frequency sub image layer, the scale factor can be adaptively adjusted according to that layer, and the adjustment factor combined with an exponential function is introduced to not only suppress high-frequency sub image noise but also preserve detail information to a great extent; using wavelet image fusion to fuse the high-frequency and low-frequency subgraphs of the input, further improving the texture details of the output image and enhancing the visual effect of the infrared image for the human eye. Reference [8] first uses NWGIF to implement multi-scale image decomposition, separating the features of a single base layer and multi-scale detail layers; Adjust the brightness of the base layer using an adaptive brightness correction model combined with defogging algorithm; Enhancing high-frequency features hidden in multi-scale detail layers using differential gain functions based on directional gradient operators; Finally, high-quality image enhancement is achieved through weighted fusion of a single base layer and multi-scale detail layers. Reference [9] first performs adaptive segmentation on the original image, generating radiation source regions and suppressing backgrounds; Estimating background radiation using morphological methods to calculate pseudo transmittance; Modulate the original image with pseudo transmittance to enhance the background and highlight the details of the radiation source; Finally, a marine infrared image enhancement algorithm based on morphological multi-scale pseudo transmittance modulation and layered fusion of radiation sources is proposed to solve the problem of excessive enhancement of edges and neighborhoods. Reference [10] first uses an adaptive modified deep network to extract features from each layer of the image, and designs a multi-scale adaptive feature fusion module (MAFM) to store and fuse multi-scale feature information from different convolutional layers; Integrating features as pixel by pixel parameter input iterative functions for image brightness enhancement; Propose a Local Feature Fusion Module (LFFM) to fuse multiple features and reconstruct images, covering both brightness enhanced images and source images; To train the entire network, a set of loss functions was designed to effectively enhance low light infrared images.

In order to improve the enhancement effect of infrared night vision images, this paper proposes an infrared night vision image enhancement algorithm based on cross-level feature fusion. Compared with the deep learning enhancement method in reference [10], this method does not solely rely on the feature extraction and fusion methods of deep networks, but emphasizes the use of cross level feature fusion strategies. This strategy can integrate different levels of information in the image more flexibly and effectively, not only ensuring better image details during the enhancement process, but also significantly reducing glare. This is the key difference in technical core and implementation effect between our method and existing deep learning enhancement methods, which can lay a more solid foundation for the subsequent processing and application of infrared night vision images.

## 2 Infrared night vision image denoising

Due to the lighting problem of infrared night vision images, the collected infrared night vision images have the problems of low contrast, difficult to distinguish the target from the background, blurring of detailed information and more noise. Therefore, denoising is needed [11–12]. In order to effectively enhance the infrared night vision images and accurately extract the effective information from the collected infrared night vision images, the first step is to improve the image contrast, increase the intensity of the detail information, and improve the visual effect of the images through the denoising process.

### 2.1 Smooth wavelet decomposition of images

First, for the acquired infrared night-vision image, and then implement the smooth wavelet decomposition of N layer gives 3N high frequency components (horizontal, vertical and diagonal directions) and one low frequency component.

During the process, the high-pass filter is set to h, the low-pass filter is g, combined with the stationary wavelet conversion principle [13–14], the wavelet component of the infrared night vision image is decomposed into a low frequency component $A_{j.k_{1},k_{2}}$ and a high frequency component $D_{j.k_{1},k_{2}}^{1}$, $D_{j.k_{1},k_{2}}^{2}$, $D_{j.k_{1},k_{2}}^{3}$ in the horizontal, vertical and diagonal directions, the process is shown in formula (1):


$$\{\begin{array}{c} {}*20cA_{j.k_{1},k_{2}}=\sum_{n_{1}}^{N}\sum_{n_{2}}^{N}h_{0}^{↑2j}\left(n_{1}-2k_{1}\right)h_{0}^{↑2j}\left(n_{2}-2k_{2}\right)A_{j-1.n_{1},n_{2}}\\ D_{j.k_{1},k_{2}}^{1}=\sum_{n_{1}}^{N}\sum_{n_{2}}^{N}h_{0}^{↑2j}\left(n_{1}-2k_{1}\right)g_{0}^{↑2j}\left(n_{2}-2k_{2}\right)A_{j-1.n_{1},n_{2}}\\ D_{j.k_{1},k_{2}}^{2}=\sum_{n_{1}}^{N}\sum_{n_{2}}^{N}g_{0}^{↑2j}\left(n_{1}-2k_{1}\right)h_{0}^{↑2j}\left(n_{2}-2k_{2}\right)A_{j-1.n_{1},n_{2}}\\ D_{j.k_{1},k_{2}}^{3}=\sum_{n_{1}}^{N}\sum_{n_{2}}^{N}g_{0}^{↑2j}\left(n_{1}-2k_{1}\right)g_{0}^{↑2j}\left(n_{2}-2k_{2}\right)A_{j-1.n_{1},n_{2}} \end{array}$$ (1)

In formula (1), k represents the image translation coefficient, j represents the scale factor.

### 2.2 Image edges as well as noise markers

After the image is decomposed by wavelet, the magnitude of the edge increases with the increase of scale, while the magnitude of the noise decreases rapidly with the increase of scale, so it is more beneficial to utilize the characteristics of the edge and the noise exhibited between adjacent scales to protect the edge while suppressing the noise. Calculate the correlation value of wavelet coefficients of neighboring scales, if the correlation is large, the pixel at that position is labeled as edge, otherwise the pixel is labeled as noise, the steps are as follows:

Step 1: For the high frequency component $D_{j}^{1}$, $D_{j}^{2}$, $D_{j}^{3}$ obtained after smooth wavelet decomposition of the infrared night vision image, for which the correlation values $CorrD_{j}^{i}$ of the expansion coefficients are calculated, and applying normalization $NcorrD_{j}^{i}$ to the correlation values, the process is shown in formula (2):


$$\left\{ \begin{array}{c} CorrD_{j}^{i}=D_{j}^{i}D_{j+1}^{i}\\ NcorrD_{j}^{i}=CorrD_{j}^{i}\frac{D_{j}^{i}}{CorrD_{j}^{i}} \end{array}\right.$$ (2)

Step 2: For different pixel points (x,y), when $NcorrD_{j}^{i}\left(x,y\right)>\left|D_{j}^{i}\left(x,y\right)\right|$, indicating that the pixel point is an edge pixel, for which edge labeling needs to be implemented, and set $CorrD_{j}^{i}\left(x,y\right)$ and $D_{j}^{i}\left(x,y\right)$ to 0.

Step 3: Thereby calculate the unlabeled edge pixel point energy $P_{j}^{i}=\frac{1}{M}\sum_{x,y}^{N}{\left[D_{j}^{i}\left(x,y\right)\right]}^{2}$ for the layer; During the process, when the calculated pixel point energy $P_{j}^{i}>\sigma_{n}^{2}$ (noise variance), then you need to go back to step 1 and restart the calculation, otherwise you need to calculate the non-zero standard deviation $\sigma_{c}$ of the value in $corrD_{j}^{i}$; If $CorrD_{j}^{i}\left(x,y\right)>3\sigma_{c}$, it means that the pixel point (x,y) is marked as an edge point.

Consistency verification is performed on the tagged pixels, i.e., if the center pixel in a region is tagged as a noise point (edge point) and most of the other pixel points in its neighborhood are tagged as edge points (noise points), then the center pixel is also tagged as an edge point (noise point). In the paper, a 3 × 3 size window is used for consistency detection. The wavelet component of the largest scale layer after image decomposition can effectively suppress the fine texture and noise, but the edge is not easy to locate, so the wavelet coefficient correlation method is not used in this layer to mark the edge and noise, but the edge points marked in the smaller scale layers are merged to mark the edges of the high-frequency component of the layer, and the unmarked points are treated as noise.

### 2.3 Neighborhood-based shrinkage of wavelet coefficients

After the pixel labeling is completed, a wavelet coefficient shrinkage algorithm that fully takes into account the nature of the pixel neighborhood is used to reduce the visual interference of the image.

During the wavelet shrinkage process [15–16], the wavelet coefficients of the decomposed infrared night vision image is set to be $D_{j}^{i}\left(x,y\right)$, thus carrying out wavelet coefficient shrinkage, the process is shown in formula (3).


$$\left\{ \begin{array}{c} newD_{j}^{i}\left(x,y\right)=\alpha \left(x,y\right)D_{j}^{i}\left(x,y\right)\\ \alpha \left(x,y\right)=\{\begin{array}{c} {}*20c1-\left(\lambda^{2}/S_{x,y}^{2}\right);\lambda^{2}/S_{x,y}^{2}<1\\ 0;\lambda^{2}/S_{x,y}^{2}>1 \end{array}\\ \lambda =\sqrt{2\sigma_{n}^{2}\mathrm{lg}L}\\ S_{x,y}^{2}=\sum_{m,n\in \varepsilon \left(x,y\right)}\left[D_{j}^{i}{\left(m,n\right)}^{2}\right] \end{array}\right.$$ (3)

In formula (3), $\sigma_{n}^{2}$ represents the image noise variance, m,n∈ε(x,y) represents the set of neighborhood points (m,n) of the pixel center (x,y), L represents the size of the image, $D_{j}^{i}\left(m,n\right)$ represents the wavelet coefficient value, $new\;D_{j}^{i}\left(x,y\right)$ represents the wavelet coefficient contraction result, α(x,y) represents the contraction factor, $\lambda^{2}/S_{x,y}^{2}$ represents the scale factor, $S_{x,y}^{2}$ represents the scale factor.

When the noise variance is large, the corresponding wavelet coefficient value of the noise may also be large, and the corresponding wavelet coefficient value becomes large after the contraction, thus generating isolated dark and bright spots, but the noise in the sub-large-scale high-frequency component is basically removed, so the following method is proposed to remove the isolated spots:

(1) For the wavelet coefficients in each of the three directions at the sub-large scale, calculate the mean mean of the absolute values of the coefficients after contraction;
(2) Set mask as a labeling matrix with size equal to the size of the high-frequency component image, if a pixel (x,y) in the sub-massive high-frequency component, the absolute value of the wavelet coefficients is less than mean, then set mask(x,y) to 1;
(3) For each pixel point (x,y) of the minimum-scale high-frequency component, if mask(x,y) is equal to 1, set the gray value of that pixel to 0.

Finally, the above method can eliminate the bright spots and dark spots in the smallest scale wavelet components, and complete the noise removal process of infrared night vision images.

## 3 Image initial enhancement

After completing the feature enhancement process of the infrared night vision image, the infrared night vision image is enhanced using the Retinex algorithm after noise removal. On this basis, after estimating the illuminance of the infrared night vision image by using the bilateral filtering method, the reflective region is enhanced by using the Sigmoid function to complete the preliminary enhancement of the infrared night vision image.

Based on Retinex theory [17–18], the infrared night vision image after noise removal is set to S(x,y), by which the illumination image R(x,y) of the image as well as the reflective image F(x,y) are obtained, the process is shown in formula (4).


S(x,y)=R(x,y)*F(x,y) (4)

In formula (4), the reflection image controls the essential properties of the image, and the illumination image determines the dynamic range that the image can achieve. The purpose of Retinex visual enhancement is to obtain the essential properties of the image from the original image, avoid the effect of illumination, and achieve the color constancy of the infrared night vision image.

The illuminance estimation takes into account the significance of the pixel brightness itself and the surrounding pixel position, and adopts the bilateral filter with the edge preservation function for the illuminance estimation, which effectively avoids the interaction between the high and low pixels near the high contrast edge during the illuminance estimation, and ultimately eliminates the “halo artifacts”.

### 3.1 Illumination estimation methods for infrared night vision images

Illumination estimation using bilateral filtering. Bilateral filtering is a very useful filtering technique that can be used in various aspects of image processing and graphics, and is a filtering technique with edge preservation. Its output value is not only related to the blank position of the surrounding pixels, but also related to their luminance difference, which is formally defined as shown in formula (5).


$$B_{s}=\frac{1}{k\left(s\right)}\sum_{p\in \Omega }^{P}f\left(p-s\right)g\left(I_{p}-I_{s}\right)I_{p}$$ (5)

In formula (5), Ω represents the set of image pixels, $B_{s}$ represents bilateral filtering output results for pixel point s, f, g represents the Gaussian function, the $I_{p}$ and $I_{s}$ represents the spatial domain value of the point as well as the luminance domain value for the pixel space p, the k(s) represents the normalization factor.

In the specific implementation process, if the bilateral filter is realized only according to the original definition, the computational efficiency is very low. For this reason, the calculation speed of bilateral filtering is improved by using the method based on the layering of gray values, which has a better effect on the calculation speed of filtering in larger space scales.

During the process, a 3D grid is used to represent the 2D gray scale image, the first two dimensions of the grid correspond to the positions of the image pixels, and the third dimension corresponds to the brightness of the image. After the definition, the bilateral filter can be calculated by the following steps:

(1) First, the infrared night vision image is regarded as a two-dimensional image, and the vector grid ψ is initialized, so that it satisfies the conditions of formula (6).

$$\psi \left(p_{x},p_{y},r\right)=\{\begin{array}{c} {}*20cI\left(p_{x},p_{y},1\right);ifr=I\left(p_{x},p_{y}\right)\\ \left(0,0,0\right);otherwise \end{array}$$ (6)

And based on the above conditions, Gaussian filtering is applied to the network and the result is shown in formula (7):


$$B\left[\psi \mathrm{\backslash rightleft}(p_{x},p_{y},r\right)=G_{\sigma_{s},\sigma_{r}}*\psi \left(p_{x},p_{y},r\right)$$ (7)

In formula (7), $\sigma_{s}$ represents the airspace parameter, $\sigma_{r}$ represents the luminance field parameter, $G_{\sigma_{s},\sigma_{r}}$ represents a Gaussian function in three dimensions.

(2) In the post-processing grid, set the final result for position $\left(p_{x},p_{y},I_{p}\right)$ is $\left(\overset{\_\_\_}{\omega I}/\omega \right)$, which completes the bilateral filtering calculation $B{\left[\psi \right]}_{p}\left(\overset{\_\_\_}{\omega I}/\omega \right)$.

The illumination image is obtained after the illumination estimation process on the input image.A part of the pixels at both ends of the histogram of the illumination image is truncated by the histogram interception method and the remaining pixels are compressed to [0,1] range, which were then corrected $y\left(i\right)=i^{\chi *i+\chi }$ using a modified Gamma correction method. When calibrating, the original image pixels are denoted by i, the control parameters are described as χ. Finally, the final illumination image y(i) is computed using the linear lazar.

### 3.2 Reflective image enhancement

After obtaining the illumination image, the reflection image can be obtained by doing the difference operation between the original image and the illumination image in the logarithmic domain. The reflection image contains the detailed information of the image, so it is very important to enhance it, and the Sigmoid function is used to enhance κ(r), the specific process is shown in formula (8):


$$\kappa \left(r\right)=\frac{2}{1+e^{-\chi *r}}-1$$ (8)

In formula (8), r represents the brightness of the reflected image, χ represents the control parameter. Where, since the reflected image brightness is in the number field of the image, there will be negative cases from time to time, and the larger the calculation result of χ, the steeper the luminance mapping curve of the reflection image is, and the more significant the enhancement of the reflection image is.

Combining the above steps, the preliminary enhancement process of infrared night image is obtained as follows:

Step 1: First, based on the infrared night vision image, obtain the image brightness image $L_{ld}$, and logarithmically, using bilateral filtering as well as gray value layering to obtain the illumination image $I_{zd}$;

Step 2: Calculate the difference of the luminance image $L_{ld}$ and illuminated image $I_{zd}$, obtain the reflection image $R_{fs}$;

Step 3: Use the histogram to intercept both ends of the illuminance image and calibrate it to determine the illuminance estimate.

Step 4: Using formula (8) for reflection image enhancement R′′, and add it to the illumination image $I_{zd}$ and get a new image New, complete the initial visual enhancement of infrared night vision images.

## 4 Image fusion enhancement based on cross-level feature fusion

After completing the preliminary visual enhancement of the image, based on the results of the preliminary visual enhancement of the image, a cross-level feature fusion network for infrared night vision image enhancement is constructed using the principle of cross-level feature adaptive fusion, the preliminary visually enhanced image is inputted into the network, and the multilevel feature extraction module of the network extracts the multi-scale features of the image at different levels, and reconstructs the features of the image at different levels using the joint feature reconstruction module. Finally, the multi-level feature fusion module is used to fuse the reconstructed multi-level features, and the model-based output effectively enhances the image feature information.

**Fig 1:**
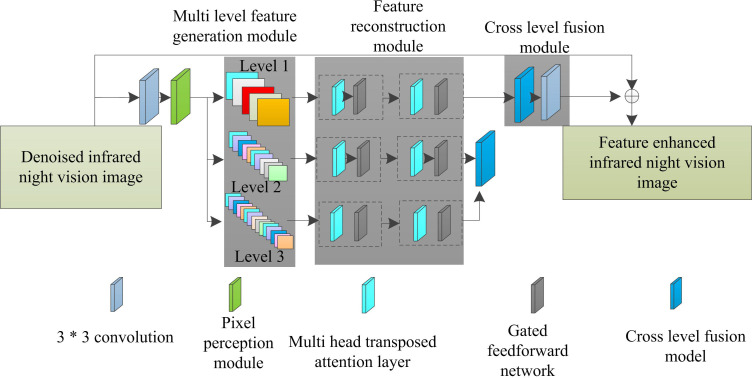
Cross-level feature fusion enhancement network for infrared night vision image enhancement.

Based on the principle of cross-level feature adaptive fusion, the specific structure of the cross-level feature fusion enhancement network constructed for infrared night vision image enhancement is shown in Fig 1.

Analyzing Fig 1, it can be seen that the multilevel feature generation module in the model employs the pixel perception module to generate multiscale feature maps at different levels (Level1, Level2, and Level3), which strengthens the expression of pixel information and improves the network adaptability. The specific operation process is as follows: the input image first enters the sampling module of the pixel perception module, which uses anti aliasing sampling to generate shallow features corresponding to different downsampling multiples. The sampling module utilizes skip connections internally to alleviate gradient dispersion problems and ensure the stability of feature information during transmission. Subsequently, the feature map undergoes point convolution, 5 * 5 depth separable convolution, and hole convolution operations in sequence. Point convolution utilizes the low computational cost of linear operations to integrate information between channels; 5 * 5 depth separable convolution expands the receptive field by sparsely connecting pixel information from a wide neighborhood without significantly increasing parameters; The 5 * 5 dilated convolution further increases the receptive field and captures a wider range of image information. Finally, the results of these three convolution operations are multiplied at the end to obtain the output of the pixel perception module, which is a multi-scale feature map of different levels. These feature maps enhance the expression of pixel information and improve the adaptability of the network to infrared night vision images. The specific feature extraction and cross-level fusion processes are as follows:

### 4.1 Multi-level feature extraction

**Fig 2:**
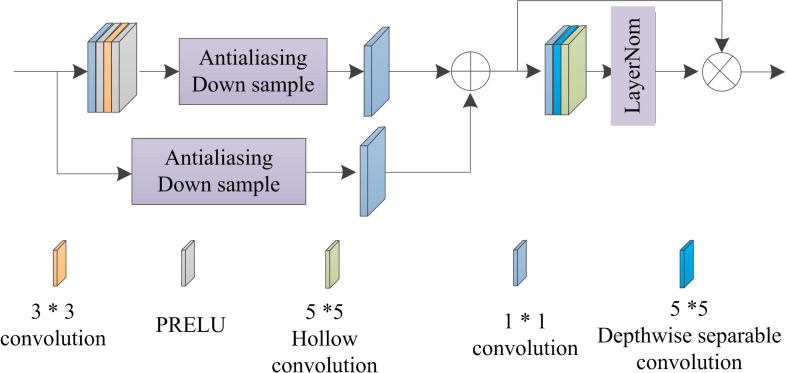
Pixel perception module structure.

Due to the overall information obscurity of infrared night vision images [19], the conventional convolutional receptive field is limited by the size of the convolutional kernel, which is unable to capture the long-distance-dependent information and impedes the extraction of neighborhood features. The pixel attention module is improved by combining the advantages of depth-separable convolution and cavity convolution, and the sensory field is effectively enlarged. In this way, a pixel-based perception is used to extract multilevel features from infrared night vision images, as shown in Fig 2.

According to the analysis of Fig 2, the input infrared night vision image is sent to the sampling module using anti aliasing sampling method. The module generates shallow feature maps through different downsampling multiples and uses skip connection mechanism internally to alleviate the gradient dispersion problem that may be caused by downsampling, ensuring stable transmission of feature information; Subsequently, the shallow feature maps generated by the sampling module enter the subsequent convolution operation process in sequence. Firstly, channel information is integrated through 1 * 1 point convolution at a lower computational cost to provide effective feature representations for subsequent operations. Then, through 5 * 5 depth separable convolution (which splits conventional convolution into deep convolution and point by point convolution, utilizing the sparse connection of wide neighborhood pixel information by large kernel convolution properties to initially expand the receptive field and capture a wider range of local features without significantly increasing parameters), and finally through 5 * 5 hole convolution (which further expands the receptive field without increasing the number of parameters, captures long-distance dependency information, and extracts richer features), point convolution, 5 * 5 depth separable convolution, and 5 * 5 hole convolution are performed. The result is a multiplication operation at the end, Effectively integrating feature information extracted by different convolution methods to obtain the final output of the pixel perception module, this module generates multi-scale feature maps of different levels (Level 1, Level 2, Level 3) through this multi form convolution operation and feature fusion method, enhances pixel information expression, and improves the network’s ability and adaptability to extract features from infrared night vision images.

It can be seen that the pixel perception module contains the sampling module and the pixel attention sub-module, both of which sequentially carry out the multilevel feature map generation and the expansion of the sensory field. Firstly, the input image flow to $\log_{2}N$ individual sampling modules to generate shallow features F corresponding to downsampling multiples. The sampling method is anti-aliased, and the sampling module utilizes hopping connections within the module to mitigate gradient dispersion.

The feature maps are sequentially subjected to point convolution, 5*5 depth separable convolution and cavity convolution, and the information between channels is integrated by using the low computational cost of linear operation, while the pixel information in a wide range of neighborhoods is sparsely connected by the nature of large kernel convolution, so as to expand the sensory field without increasing the parameters dramatically. Finally, the results of multi-form convolution operation are combined with F at the end to obtain the module output Y. Given the input X, the process is shown in formula (9):


$$\left\{ \begin{array}{c} F_{shaw}=Conv_{1\times 1}\left(\Phi \left(Conv_{3\times 3}\left(Conv_{1\times 1}\left(X\right)\right)\right)\right)+Conv_{1\times 1}\left(\Phi \left(X\right)\right)\\ Y=\left[DConv_{5\times 5}\left(DWConv_{5\times 5}\left(Conv_{1\times 1}\left(F^{\log_{2}N}\right)\right)\right)\right]\cdot F^{\log_{2}N} \end{array}\right.$$ (9)

In formula (9), Φ represents sawtooth sampling. N represents the sampling multiplier. $Conv_{1\times 1}$ represents the 1*1 convolution, $Conv_{3\times 3}$ represents the 3*3 convolution, $DConv_{5\times 5}$, $DWConv_{5\times 5}$ represents a 5*5 depth separable convolution as well as a 5*5 null convolution.

### 4.2 Multi-level infrared night vision image feature reconstruction

Due to the redundancy of useless information and the weakening of the characterization ability of effective information in infrared night vision images, the interaction of feature information within the image is limited, which is not conducive to the reconstruction of image details. In order to filter the redundant information and establish the long-term dependence between local and global features, an efficient multi-head transposed attention module is introduced to model the global information and realize the feature reconstruction. The module consists of a multihead transposed attention layer (MDTA) and a gated feedforward network (GDFN).

**Fig 3:**
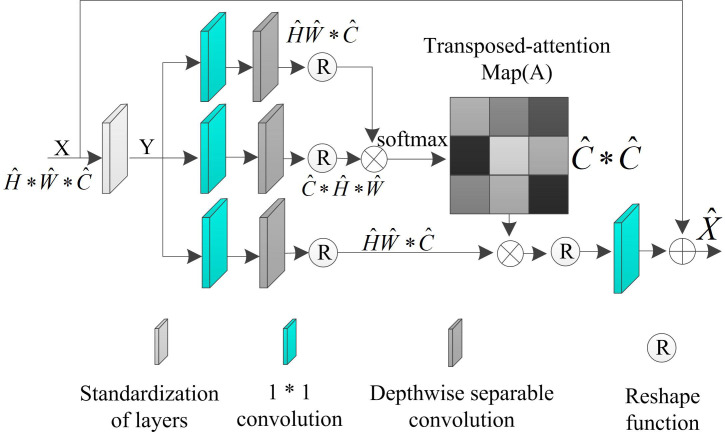
Multi-head transposition attention layer (MDTA) structure.

The MDTA structure in the module is shown in Fig 3.

Analysis of Fig 3 shows that, given a tensor input normalized by the layers of the normalization is $Y\in R^{\hat{H}*\hat{W}*\hat{C}}$. By aggregating the image context information between the channels through 1*1 convolution, followed by encoding the spatial context information between the channels through 3*3 depth-separated convolution, both of which utilize the complementary advantages of convolution operations to strengthen the local spatial characterization capability, and thus generate queries Q, index K as well as the key value V. Finally, applying the dot product operation to reshape the projection of Q and K, Q and K interactions generate a transposed attention map A with size $R^{\hat{C}*\hat{C}}$, implicitly modeling the global relationship between image cables, implicitly model the global relationship between the image cords. The MDTA module flow is shown in formula (10):


$$\left\{ \begin{array}{c} Attention\left(\hat{Q},\hat{K},\hat{V}\right)=\hat{V}\cdot Soft\max\left(\hat{K}\cdot \hat{Q}/\beta \right)\\ \hat{X}=W_{p}Attention\left(\hat{Q},\hat{K},\hat{V}\right)+X \end{array}\right.$$ (10)

In formula (10), X and $\hat{X}$ represents the input and output feature images, $\left(\hat{Q},\hat{K},\hat{V}\right)$ represents the decomposition result of the input $Y\in R^{\hat{H}*\hat{W}*\hat{C}}$ of the original image X, β represents the point product size parameter controlling Q and K.

**Fig 4:**
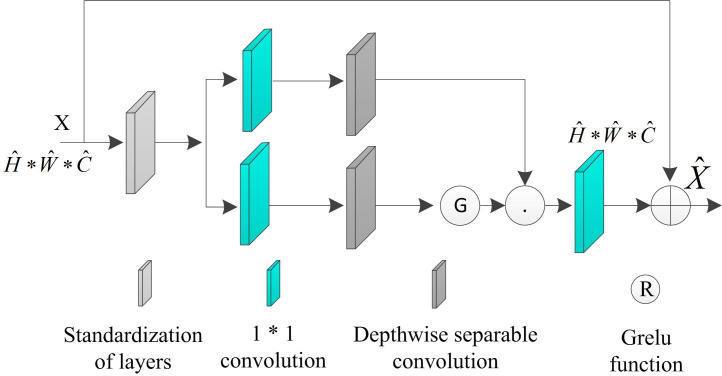
Gated Feedforward Network (GDFN) Structure.

The structure of GDFN in the module is shown in Fig 4. GDFN is improved according to the conventional feed-forward network, which mainly introduces an additional gating mechanism in the conventional feed-forward network and replaces the conventional convolution with the depth-separable convolution.

The gating mechanism [20] in Fig 4 is represented as the product of two parallel paths of linear transformation layers, one of which invokes the CELU activation function to realize the control information flow. The similarity with MDTA is that GDFN also uses depth-separable convolutional encoding of neighborhood pixel position information to optimize the linkage of spatial context information, which helps to recover the image structure. Given a tensor input of $X\in R^{\hat{H}*\hat{W}*\hat{C}}$, the GDFN training process is shown in formula (11):


$$\{\begin{array}{c} {}*20c\hat{X}=W_{p}^{0}Gating\left(X\right)+X\\ Gating\left(X\right)=\varphi \left(W_{d}^{1},W_{p}^{1}\left(LN\left(X\right)\right)\right)⊕W_{d}^{1},W_{p}^{1}\left(LN\left(X\right)\right) \end{array}$$ (11)

In formula (11), φ represents the GELU function, LN represents layer standardization. ⊕ represents the element-by-element cumulative multiplication symbol.

The difference with MDTA is that GDFN achieves the purpose of information interaction and complementarity by controlling the flow of information at each level in the branch, while MDTA utilizes parallel structure and multiple forms of convolution to enrich the contextual information.

### 4.3 Adaptive cross-level feature fusion

**Fig 5:**
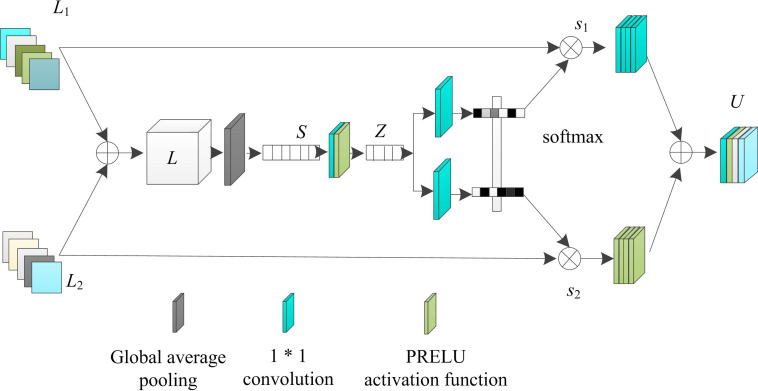
Structure of Adaptive Feature Fusion Module (SKFF).

The commonly used feature fusion methods for multi-scale networks are mainly splicing and summing, which are characterized by simple and efficient operation, but the operation limits the feature expression ability of the network, which is not conducive to the improvement of model performance. In order to fully utilize the high quality features extracted from the network, an adaptive feature fusion module (SKFF) is introduced into the network framework of this paper, which utilizes the self-attention mechanism to nonlinearly fuse the features of different levels, and the structure of the module is shown in Fig 5.

Analyzing Fig 5 shows that SKFF can be regarded as two stages. The first stage is the feature aggregation stage: Firstly, the tensors are merged sequentially using splicing, reshaping and accumulation operations, and during the process, set $L=L_{1}+L_{2}$; Second, the features are aggregated from the spatial dimension using global average pooling $L\in R^{H\times W\times C}$ get the channel information $s\in R^{1\times 1\times C}$. Finally, point-by-point convolution is used to strengthen the feature correlation and generate the tensor $z\in R^{1\times 1\times r}$, where the 1/8th of convolution C is r. The second stage is the weighted fusion stage; The tensor z is transmitted in two forward directions. The feature descriptors $v_{1}$ and $v_{2}$. $\left(v_{1},v_{2}\in R^{1\times 1\times C}\right)$ are generated by two forward propagation and 1*1 convolution respectively; Followed by the computation of the attention weights $s_{1}$, $s_{2}$ using the softmax function. Finally $L_{1}$ and $L_{2}$ are multiplied with the attention weights, respectively, and summing at the end to get the module output. The specific flow is shown in formula (12).


$$U=L_{1}s_{1}+L_{2}s_{2}$$ (12)

The adaptive feature fusion module (SKFF) plays a key role in the network framework of this article. It uses self attention mechanism to nonlinearly fuse different hierarchical features, avoiding the limitations of traditional feature fusion methods such as concatenation and summation, which limit the network’s feature expression ability and are not conducive to improving model performance due to their simple operation. Consider this module as two stages of feature aggregation and weighted fusion. Firstly, features are aggregated and correlated through operations such as concatenation, reshaping, accumulation, global average pooling, and point by point convolution. Then, feature descriptors are generated through 1*1 convolution. The softmax function is used to calculate attention weights and weight the sum to obtain the output. This design conforms to the cutting-edge concept of multi-scale feature fusion and can dynamically weight features at different levels to improve the effectiveness of feature fusion.

### 4.4 The loss function

The Charbonnier loss function is widely used in the field of image restoration due to its own operational boundaries and convergence properties. The Charbonnier loss function is formulated as shown in formula (13).


$$L_{con}=\sqrt{{\left(I_{bq}-I_{yb}\right)}^{2}+\varepsilon^{2}}$$ (13)

In formula (13). $I_{bq}$, $I_{yb}$ represents loss function processing of infrared night vision image labels as well as samples, ε represents a constant.

However, the Charbonnier loss function only calculates the error between pixels and does not take the global information into account, so the problem of excessive smoothing usually occurs. In order to further enhance the realism of the high-frequency details of the image while recovering the image, an edge loss function is attached to the Charbonnier loss function for constraining the high-frequency components between labels and samples, as shown in formula (14).


$$L_{edge}=\sqrt{{\left(Lap\left(I_{bq}\right)-Lap\left(I_{yb}\right)\right)}^{2}+\varepsilon^{2}}$$ (14)

In formula (14), $Lap\left(I_{bq}\right)$, $Lap\left(I_{yb}\right)$ represent the edge features extracted by the Laplacian operator Lap versus $I_{bq}$, $I_{yb}$.

The combination of the above loss functions has good efficacy in the image recovery task, but has limited ability to recover implicit information such as image structure. In order to further improve the visual effect of the image, the structural similarity loss function $L_{SSIM}$ is introduced, and auxiliary for model optimized in the directions of contrast and structure. In summary, the combination of the three is constructed into a joint loss function as shown in formula (15):


$$Loss=L_{con}\left(X,Y\right)+a\cdot L_{edge}\left(X,Y\right)+L_{SSIM}\left(X,Y\right)$$ (15)

According to the loss function to complete the infrared night vision image feature enhancement output results, to realize the infrared night vision image feature enhancement processing.

## 5 Experiment

The infrared night vision image enhancement algorithm based on cross-level feature fusion (proposed method), the infrared image enhancement algorithm based on low-frequency redistribution and edge enhancement from reference [5] (comparison method 1), the infrared image enhancement algorithm based on adaptive multi-feature fusion from reference [6] (comparison method 2), the infrared image fusion enhancement algorithm based on the improved wavelet thresholding function and full-scale Retinex from reference [7] (comparison method 3), and Reference [8] Multi scale infrared image enhancement method based on non-uniform weighted guided filtering (comparative method 4) are selected for comparative testing, to verify the feasibility of the proposed method in the process of infrared night vision image enhancement in the practical application.

### 5.1 Experimental setup

**Fig 6:**
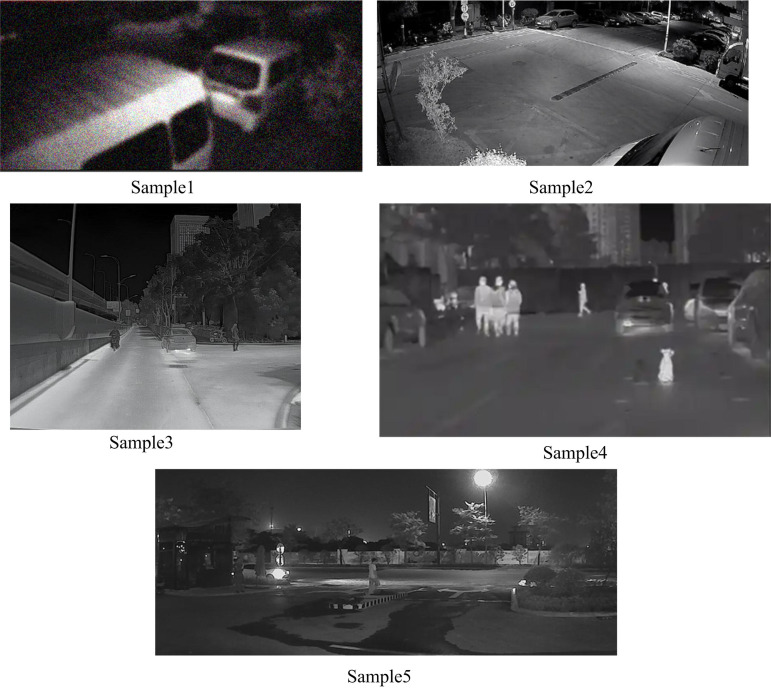
Infrared night vision image test sample.

During the experiment, an open field monitoring point was selected as the test site, and an InGaAs uncooled short-wave infrared camera was used to collect infrared night-vision images at different locations of the site. The collected infrared night vision images were integrated to create a collection of test samples for experimental use. At the same time, Ubuntu 20.04 LTS operating system, Intel Core i7-10700K CPU, Python 3.8 programming language, and OpenCV image processing library were selected to test the image enhancement effect. Based on the test sample set, five images are selected as test samples to test the effectiveness of the image enhancement method. The specific test samples are shown in Fig 6.

In Fig 6, there is no issue of inaccurate focusing in samples 1 and 4. Sample 1: Due to the object being close to the camera and in a low light environment in the shooting scene, the contour edges of the object exhibit a certain degree of blur, which is a characteristic of shortwave infrared imaging under complex lighting conditions and not a focusing error; In sample 4, the overall contrast of the image is low and there is some noise interference, making the image appear unclear. However, after analyzing the camera shooting parameters and imaging principles, this performance is also caused by infrared imaging characteristics and environmental factors, rather than inaccurate focusing.

The relevant parameter settings during the experiment are shown in Table 1.

**Table 1:** Relevant parameters during the experimental process.

Project		Parameterization
InGaAs uncooled short-wave infrared camera	Sensor type	Sony IMX990
	Sensor Scale	1280*1024
	Image element size	5μm*5μm
	Spectral response	400-1800nm
	Gain range	1-15x
	Sensitivity	NETD < 50mK
	Frame rate	25Hz
	Measurement accuracy	±2%
	Long infrared fluctuations	940nm
Cross-level feature convergence enhancement networks	Learning rates	0.001
	Batch size	32
	Number of training rounds	50
Smooth wavelet decomposition	Number of layers	4
Bilateral filter	Spatial parameters	15
	Brightness parameter	0.1

In this experiment, a detailed hyperparameter analysis was conducted on the proposed infrared night vision image enhancement algorithm based on cross level feature fusion. The learning rate of the cross level feature fusion enhanced network is set to 0.001, which can balance the convergence speed and stability well during the training process; Set the batch size to 32, which ensures training efficiency while also allowing the model to fully learn the diversity of data; The number of training rounds is set to 50, and after multiple experimental verifications, the model can achieve good convergence results under this number of rounds. Setting the number of smooth wavelet decomposition layers to 4 can effectively extract feature information from images at different scales. The spatial parameter of bilateral filtering is set to 15, and the brightness parameter is set to 0.1. By adjusting these two parameters, the image edge details are well preserved while denoising. The reasonable setting of these hyperparameters plays a key role in achieving good performance of the algorithm in infrared night vision image enhancement tasks.

In this experiment, in order to ensure the fairness and effectiveness of the comparative testing, the optimal experimental parameters that each comparative method can achieve in the experimental environment were selected through multiple experiments and parameter adjustments for the four comparative methods under the conditions of this experiment, in order to verify the practical feasibility of the proposed method in the process of enhancing infrared night vision images.

### 5.2 Analysis of test results

**Fig 7:**
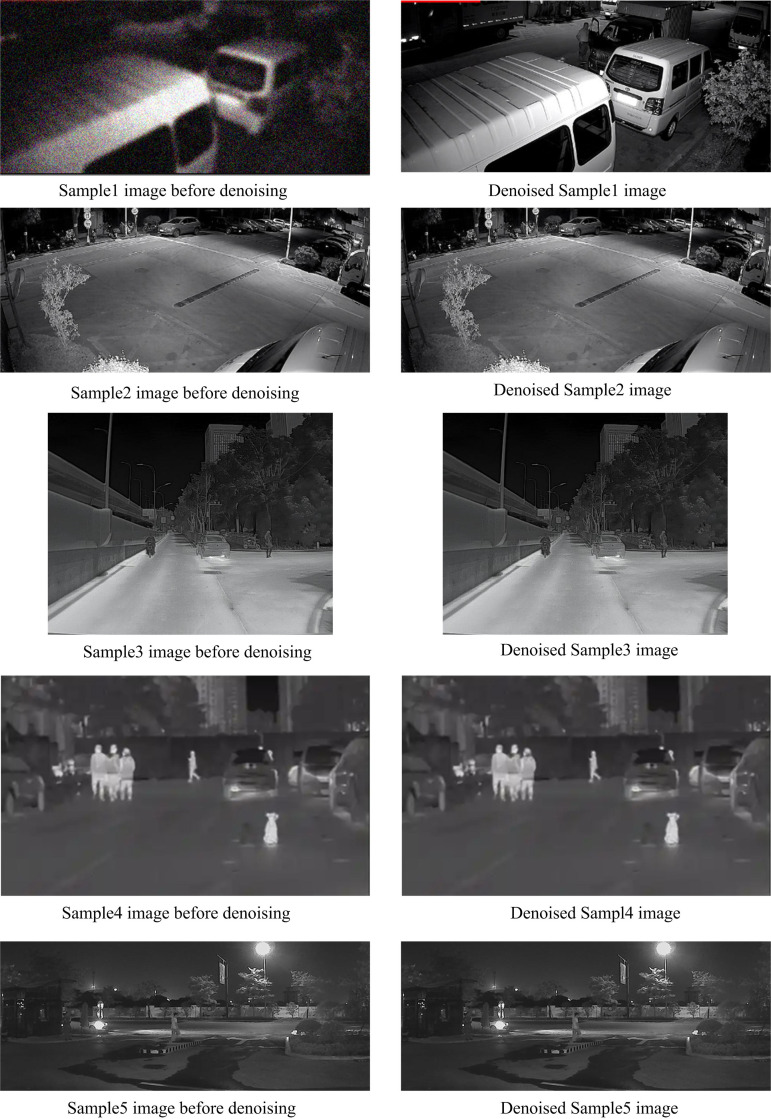
Comparison of proposed method before and after denoising.

We conducted infrared night vision image enhancement in the test samples and tested the image denoising effect of the proposed method. The test results are shown in Fig 7.

From the analysis of Fig 7, it can be seen that the proposed method for enhancing infrared night vision images is more conducive to suppressing noise while protecting edges and labeling pixel edges, as it utilizes the features exhibited by edges and noise between adjacent scales during infrared night vision image denoising. Therefore, this method preserves the detailed information within the image completely during infrared night vision image denoising. This proves that the proposed method is effective in enhancing infrared night vision images.

After completing the detection of image denoising effect, the proposed method, comparison method 1, comparison method 2, comparison method 3 and comparison method 4 were used to continue the infrared night vision image enhancement process, and the peak signal-to-noise ratio and structural similarity were selected as the performance test indexes of image enhancement to test the actual enhancement performance of the image enhancement process of the different methods, the specific peak signal-to-noise ratio, the peak signal-to-noise ratio, the peak signal-to-noise ratio, the peak signal-to-noise ratio, the peak signal-to-noise ratio, the peak signal-to-noise ratio, the peak signal-to-noise ratio, and the acquisition process for the structure similarity ratio PSNR and structural similarity SSIM is shown in formula (16):


$$\{\begin{array}{c} {}*20cPSNR=10\times \log_{10}\left(\frac{\max\left(p_{i,j}\right)}{MSE}\right)\\ SSIM\left(x,y\right)=\frac{\left(2\mu_{x}\mu_{y}+\zeta_{1}\right)\times \left(2\sigma_{xy}+\zeta_{2}\right)}{\left(\mu_{x}^{2}+\mu_{y}^{2}+\zeta_{1}\right)\times \left(\sigma_{x}^{2}+\sigma_{y}^{2}+\zeta_{2}\right)} \end{array}$$ (16)

In formula (16), $\zeta_{1}$, $\zeta_{2}$ represents constant. $\sigma_{x}^{2}$, $\sigma_{y}^{2}$ represents the contrast variance of the image x and image y. $\mu_{x}$, $\mu_{y}$ represents the mean luminance variance. $\sigma_{xy}$ represents the covariance between $\mu_{x}$ and $\mu_{y}$. MSE represents the mean square error between the original image and the processed image. $\max\left(p_{i,j}\right)$ represents the maximum possible pixel value in the image.

The higher the PSNR as well as SSIM values during image enhancement, the higher the structural similarity between the processed image and the original image, i.e., the better the image quality. The specific test results are shown in Table 2.

**Table 2:** Test results of actual infrared night vision image enhancement performance using different methods.

Test samples	Test indicators	Actual infrared night vision image enhancement performance test results				
		The proposed methodology	Method of comparison 1	Method of comparison 2	Method of comparison 3	Method of comparison 4
1	PSNR/dB	35	33	31	28	25
	SSIM	0.87	0.81	0.78	0.75	0.71
2	PSNR/dB	38	36	35	31	29
	SSIM	0.82	0.80	0.75	0.77	0.74
3	PSNR/dB	33	30	28	26	23
	SSIM	0.88	0.84	0.78	0.73	0.69
4	PSNR/dB	30	25	19	18	14
	SSIM	0.79	0.74	0.71	0.66	0.67
5	PSNR/dB	36	33	35	25	21
	SSIM	0.73	0.68	0.66	0.63	0.58

Analyzing Table 2, it can be seen that the peak signal-to-noise ratio and the structural similarity of the detected infrared night vision image after infrared night vision image enhancement by the proposed method are the best among the five methods. This is mainly due to the fact that the proposed method uses bilateral filtering algorithm to further enhance the illumination of the feature-enhanced infrared night-vision image during the image enhancement, and thus the proposed method is effective in infrared night-vision image enhancement.

**Fig 8:**
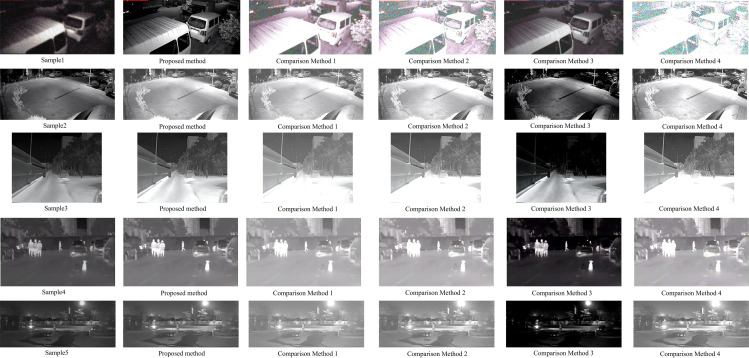
Actual image enhancement effect test results of different methods.

Based on the above test results, the proposed method, comparison method 1, comparison method 2, comparison method 3 and comparison method 4 are used to carry out infrared night vision image enhancement, and the actual enhancement effect of different methods is tested, and the test results are shown in Fig 8.

Analyzing Fig 8, we can see that, when carrying out infrared image enhancement, comparing method 1 due to the uniform redistribution of low-frequency images, increasing the resource consumption of the algorithm, so the method in the infrared image enhancement, algorithmic performance is poor; comparing method 2 due to the joint image pyramid fusion features, feature parameters of the mismatch between the algorithms, resulting in the use of the algorithm to increase the energy consumption, so the method in the infrared image enhancement, algorithmic performance Comparison method 3 has poor enhancement effect in infrared image enhancement due to the existence of multi-degree fusion when fusing the input high-frequency and low-frequency subgraphs using wavelet image fusion; Comparison method 4 has poor enhancement effect in infrared image enhancement due to the fact that a part of the foreground target image information is segmented into the background image when region segmentation is performed; Because the bilateral filtering algorithm is used to implement further illumination enhancement to the feature-enhanced infrared night vision image during image enhancement, the proposed method has good enhancement effect when infrared night vision image enhancement is carried out.

To comprehensively evaluate the effectiveness of the infrared night vision image enhancement algorithm based on cross level feature fusion proposed in this article, public datasets FLIR and KAIST were added in the ablation experiment, which contain rich infrared night vision images and can more comprehensively evaluate the performance of the proposed method. The setting only uses denoising and preliminary enhancement steps, does not include cross level feature fusion network as the basic method, adds denoising and preliminary enhancement steps to the basic method as denoising+preliminary enhancement, only uses cross level feature fusion network for image enhancement as a cross level feature fusion network, and includes denoising, preliminary enhancement, and cross level feature fusion network as the complete algorithm. Meanwhile, citing time consumption as an evaluation metric. The experimental results are shown in Table 3.

**Table 3:** Results of ablation experiments on different datasets.

Method	FLIR dataset time consumption (ms)	KAIST dataset time consumption (ms)
Base method	150	160
Denoising+preliminary enhancement	120	130
Cross level feature fusion network	80	85
Complete method	50	55

According to the analysis in Table 3, it can be seen that the complete method has the shortest time consumption on the FLIR and KAIST datasets, which are 50ms and 55ms respectively. This is because in the implementation process of the complete method, the bilateral filtering and other operations were optimized based on grayscale value layering, effectively improving the calculation speed. Especially at large spatial scales, this optimization has a more significant effect on improving the filtering calculation speed, making the overall processing time shorter even if the complete method contains more complex steps. The time consumption of the denoising+preliminary enhancement method is reduced compared to the basic method, indicating a certain optimization synergy between the denoising processing and preliminary enhancement steps in implementation. When used alone, the cross level feature fusion network consumes relatively less time, indicating that the network structure itself has certain advantages in computational efficiency. Overall, the experimental results not only validate the effectiveness of the algorithm proposed in this paper in terms of performance, but also indirectly reflect the positive effect of the grayscale value based hierarchical optimization strategy on improving the processing speed of the algorithm.

## 6 Conclusion

With the gradual increase in the use of infrared images in the field of night surveillance, the implementation of visual enhancement of infrared night vision images is particularly important. Aiming at the problems existing in the traditional enhancement methods, an infrared night vision image enhancement algorithm based on cross-level feature fusion is proposed. Based on the image denoising results, the method constructs a cross-level fusion feature enhancement network to extract image features, and fuses the images across levels; finally, the Retinex bilateral filtering is used to implement further illumination enhancement on the enhanced image to improve the visual effect and complete the image enhancement process. This method is more complicated when extracting image features, and we will continue to optimize the enhancement method for this problem in the future.
